# Supplementary material for: High performance artificial visual perception and recognition with a plasmon-enhanced 2D material neural network
Source: Nat Commun. 2024 Mar 19;15:2471. doi: 10.1038/s41467-024-46867-8 (PMC10951348; doi:10.1038/s41467-024-46867-8)
Supplement: Supplementary file 1 — Supplementary Information [file 41467_2024_46867_MOESM1_ESM.pdf]

# Supplementary Information

# High performance artificial visual perception and recognition with a plasmon-enhanced 2D material neural network

Tian Zhang[1], Xin Guo[1,2], Pan Wang[1,2], Xinyi Fan[1], Zichen Wang[1], Yan Tong[1], Decheng Wang[1], Limin Tong[1,2] & Linjun Li[1,2]*

**[1] State Key Laboratory of Extreme Photonics and Instrumentation, College of Optical Science and Engineering, Zhejiang University, Hangzhou 310027, China**

**[2] Intelligent Optics and Photonics Research Center, Jiaxing Institute Zhejiang University, Jiaxing, China**

*Email: lilinjun@zju.edu.cn

**Table of content**

**Supplementary Fig. 1 |** Schematics of the human visual system. ....3
**Supplementary Fig. 2 |** Schematic fabrication process of the device. ....4
**Supplementary Fig. 3 |** Full SEM view of the PPTA. ....5
**Supplementary Fig. 4 |** Circuit of the ANN PPTA. ....6
**Supplementary Fig. 5 |** Characterization of $MoS_2$, h-BN and $WSe_2$. ....7
**Supplementary Fig. 6 |** Comparison of photocurrent in 2D PPT with and without Ag nanogratings. ....8
**Supplementary Fig. 7 |** Photothermoelectric effect of 2D PPT with and without Ag nanogratings. ....9
**Supplementary Fig. 8 |** Optical setup. ....10
**Supplementary Fig. 9 |** Experimental setup. ....11
**Supplementary Fig. 10 |** Measurement of optoelectronic characteristics of 2D PPT. ....12
**Supplementary Fig. 11 |** PPTA uniformity. ....13
**Supplementary Fig. 12 |** Measurement of optoelectronic characteristics of 2D PPT. ....14
**Supplementary Fig. 13 |** Implementation of PPT photoresponsivity. ....15
**Supplementary Fig. 14 |** The training processes of the ANN with experimental photoresponsivity. ....16
**Supplementary Fig. 15 |** Photoresponsivity and weight distributions of the array. ....17
**Supplementary Fig. 16 |** Transfer characteristic curves. ....17
**Supplementary Fig. 17 |** Training datasets. ....18
**Supplementary Fig. 18 |** Time-resolved measurements. ....19
**Supplementary Fig. 19 |** Ultrafast image recognition. ....20
**Supplementary Fig. 20 |** Discussion on the limited speed of device operation. ....21
**Supplementary Fig. 21 |** Plasmon-enhanced photodetector with adjustable photoresponsivity. ....22
**Supplementary Fig. 22 |** Schematic of the mechanism of the 2D PPT device with ultra-high DR. ....23
**Supplementary Fig. 23 |** Transfer characteristic curve and its application in image pre-processing. ....24
**Supplementary Table 1 |** Summary of the performance of individual plasmonic phototransistor. ....24
**Supplementary Table 2 |** Comparison of the proposed neuromorphic device with previous report. ....25
**Supplementary Note 1 |** The reason and mechanism of the device with ultra-high DR. ....26
**Supplementary Note 2 |** An analysis on the performance of the individual plasmonic phototransistor. ....27
**Supplementary References**. ....29

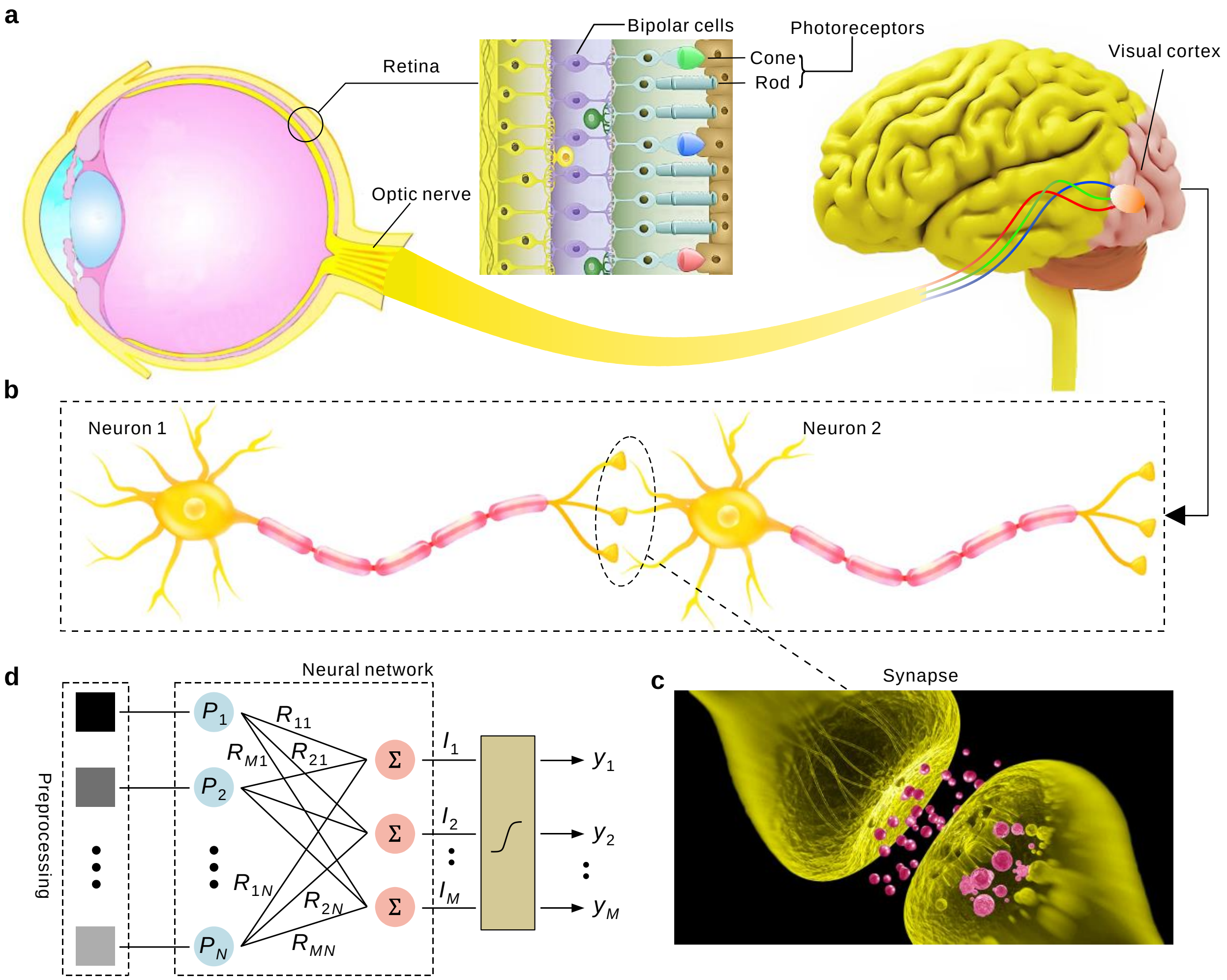


**Supplementary Figure 1: Schematics of the human visual system. a**, Illustration of an neuromorphic visual system. A biological visual system consisting of the retina (sensing and pre-processing), optic nerve (transducing) and the visual cortex (image recognition). The inset shows a magnified view of the retina organized in a hierarchical way. Photoreceptors (cone and rod cells) are used to sense external color optical information, and the information can be pre-processed through ON and OFF bipolar cells[1]. **b**, Schematic diagram of a neural network formed by synaptic connections between neurons. **c**, Diagram of neurotransmitter transmission between synapses. **d**, Schematic of the classifier where the preprocessed signal is used as input.

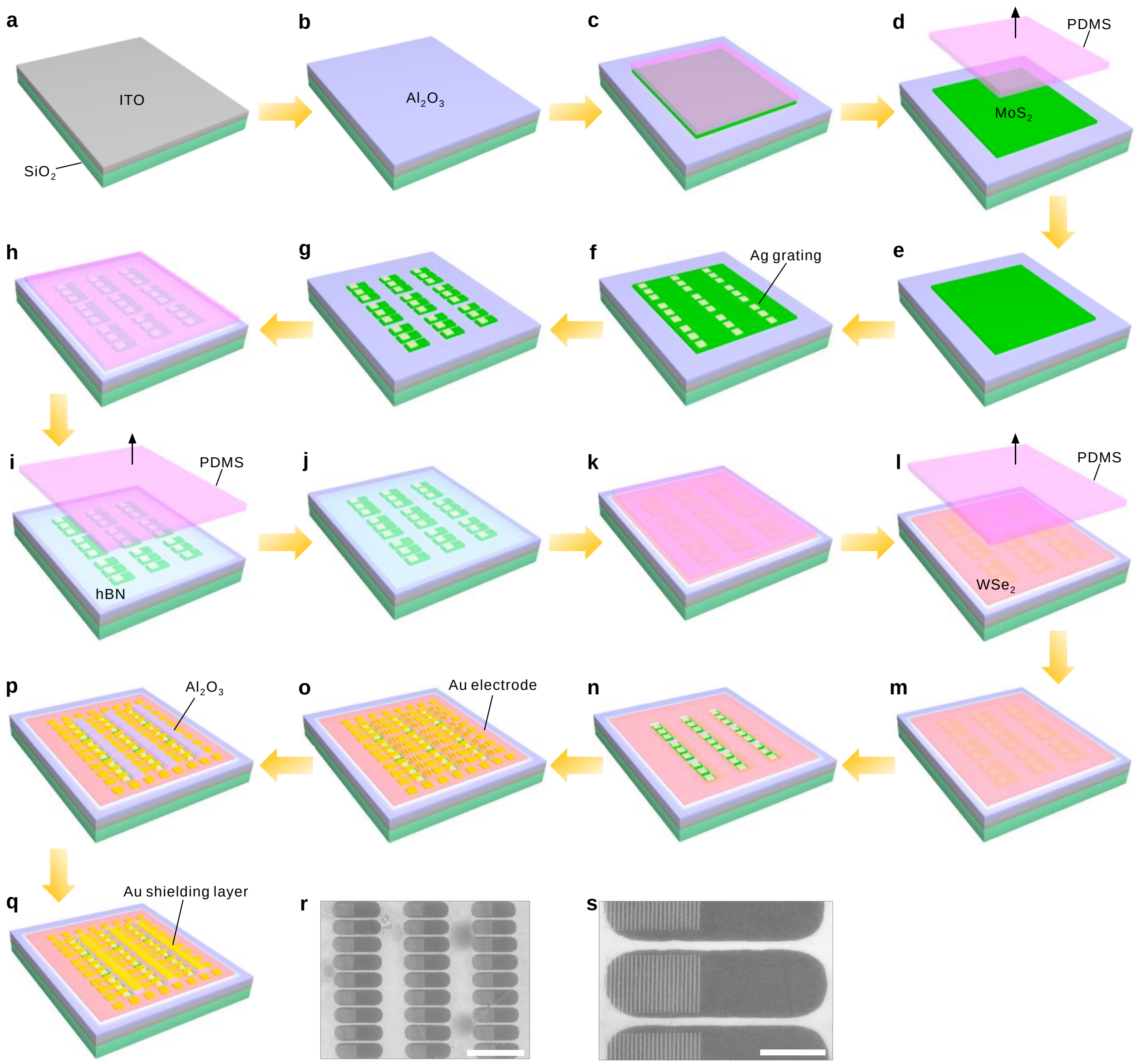


**Supplementary Figure 2: Schematic fabrication process of the device.** **a**, Sputtering a layer of indium tin oxide (ITO) on $SiO_2$ substrate by magnetron sputtering. **b**, Growth of a thin film of $Al_2O_3$ using atomic layer deposition technology. **c**, An $MoS_2$ flake is first transferred to a transparent PDMS film by mechanical exfoliation method. **d**, **e**, After the PDMS is lifted off, the $MoS_2$ is left on the substrate just like stamping a pattern with a stamp. **f**, Fabrication of Ag nanograting on $MoS_2$ flake using EBL through overlay process. **g**, RIE of the $MoS_2$ flake using a mask made of EBL. **h**, **i**, **j**, Using the same method, an h-BN flake is transferred on the $MoS_2$ flake. **k**, **l**, **m**, With the same method, a $WSe_2$ flake is transferred on the h-BN/$MoS_2$ flake. **n**, RIE of h-BN/ $WSe_2$ flakes using an EBL mask exposes the Ag nanograting to air. **o**, EBL is used

to define Au/Cr electrodes on the obtained heterostructures. **p**, **q**, Finally, the $Al_2O_3$ and Cr/Au layers are sputtered sequentially on the EBL-defined pattern. **r**, SEM view of the sample during the (g) processing step. Scale bar, 20 μm. **s**, Enlarged SEM view of the sample in (r). Scale bar, 5 μm.

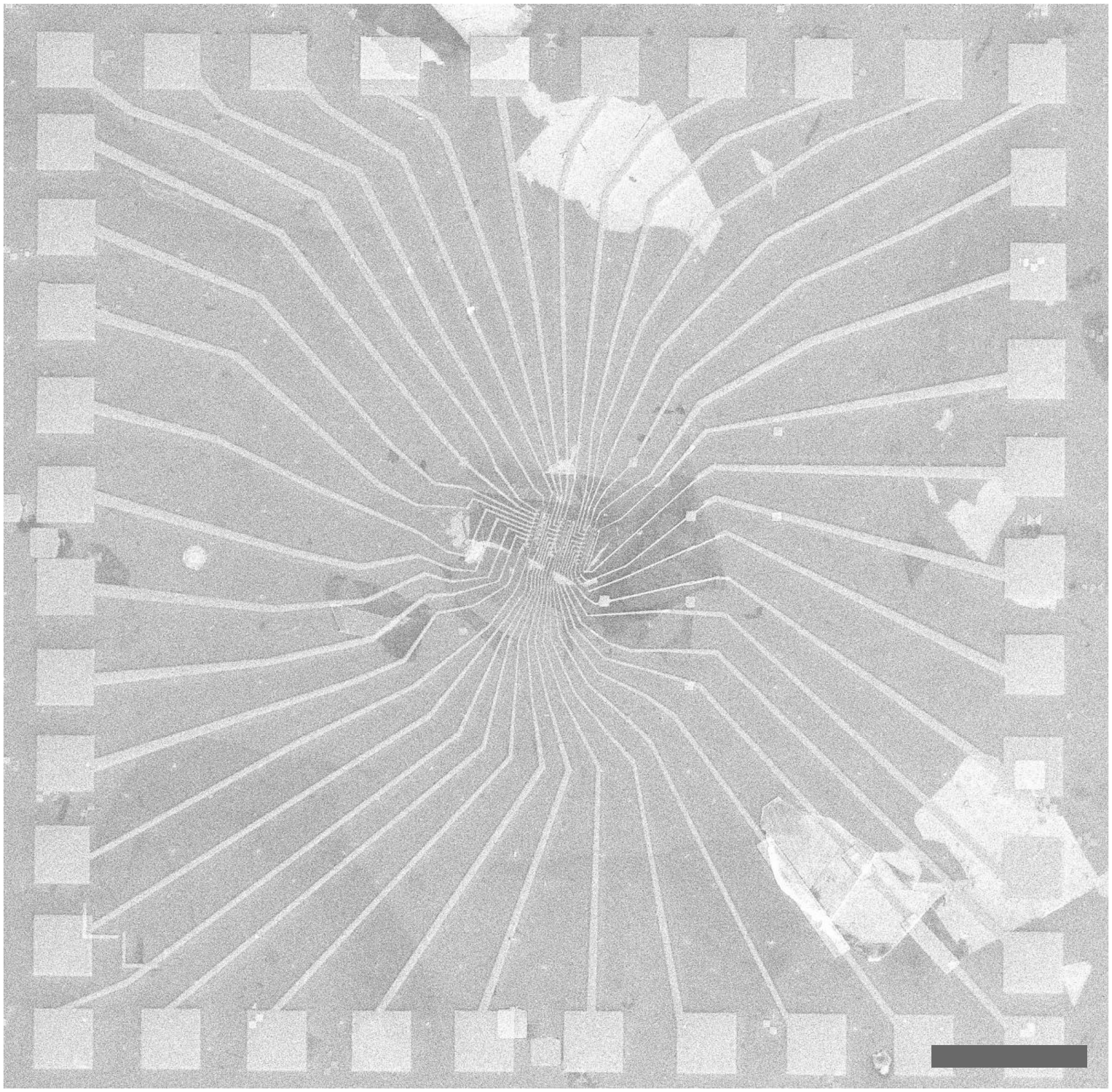

**Supplementary Figure 3: Full SEM view of the PPTA. Scale bar, 200 μm.**

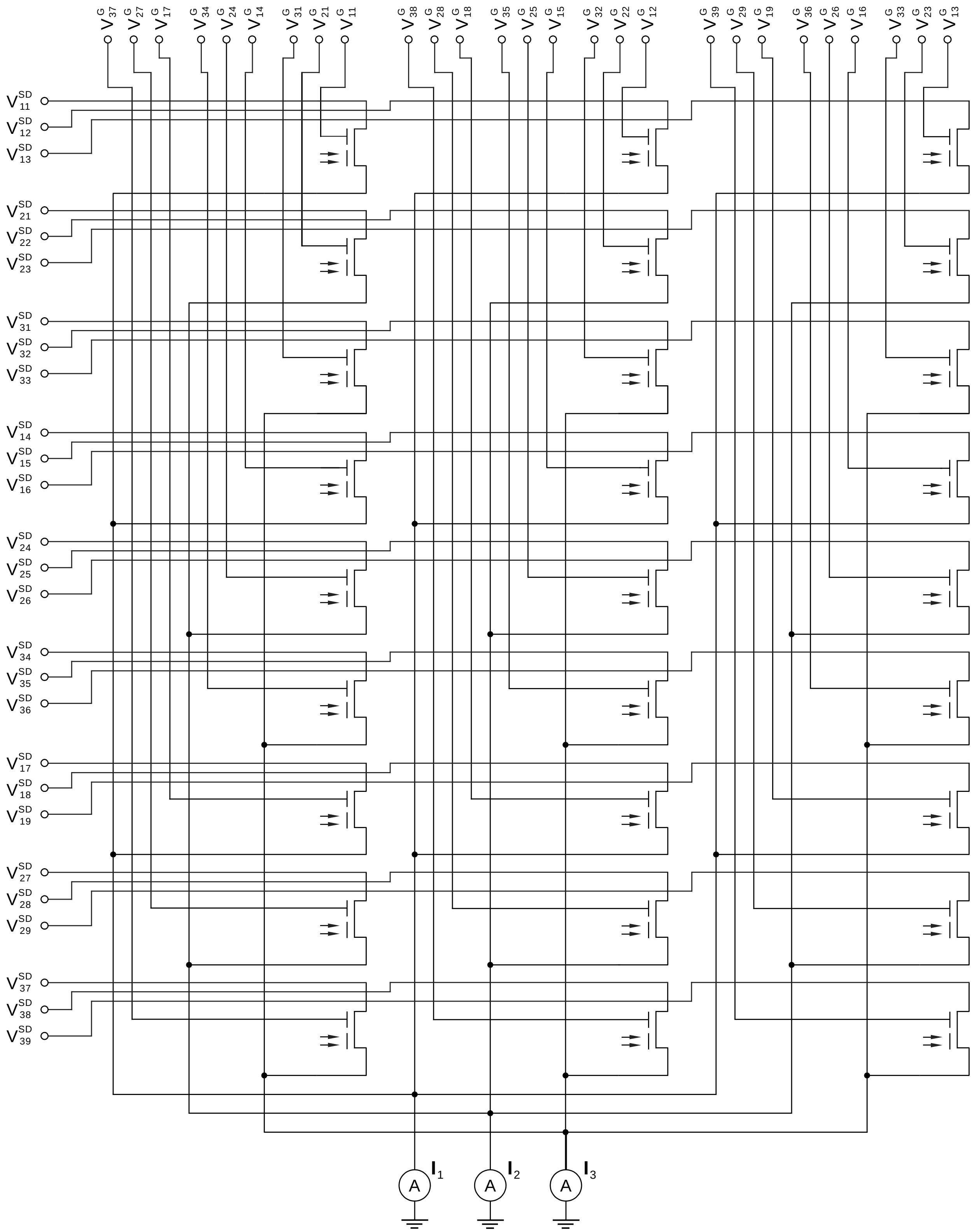


**Supplementary Figure 4: Circuit of the ANN PPTA.**

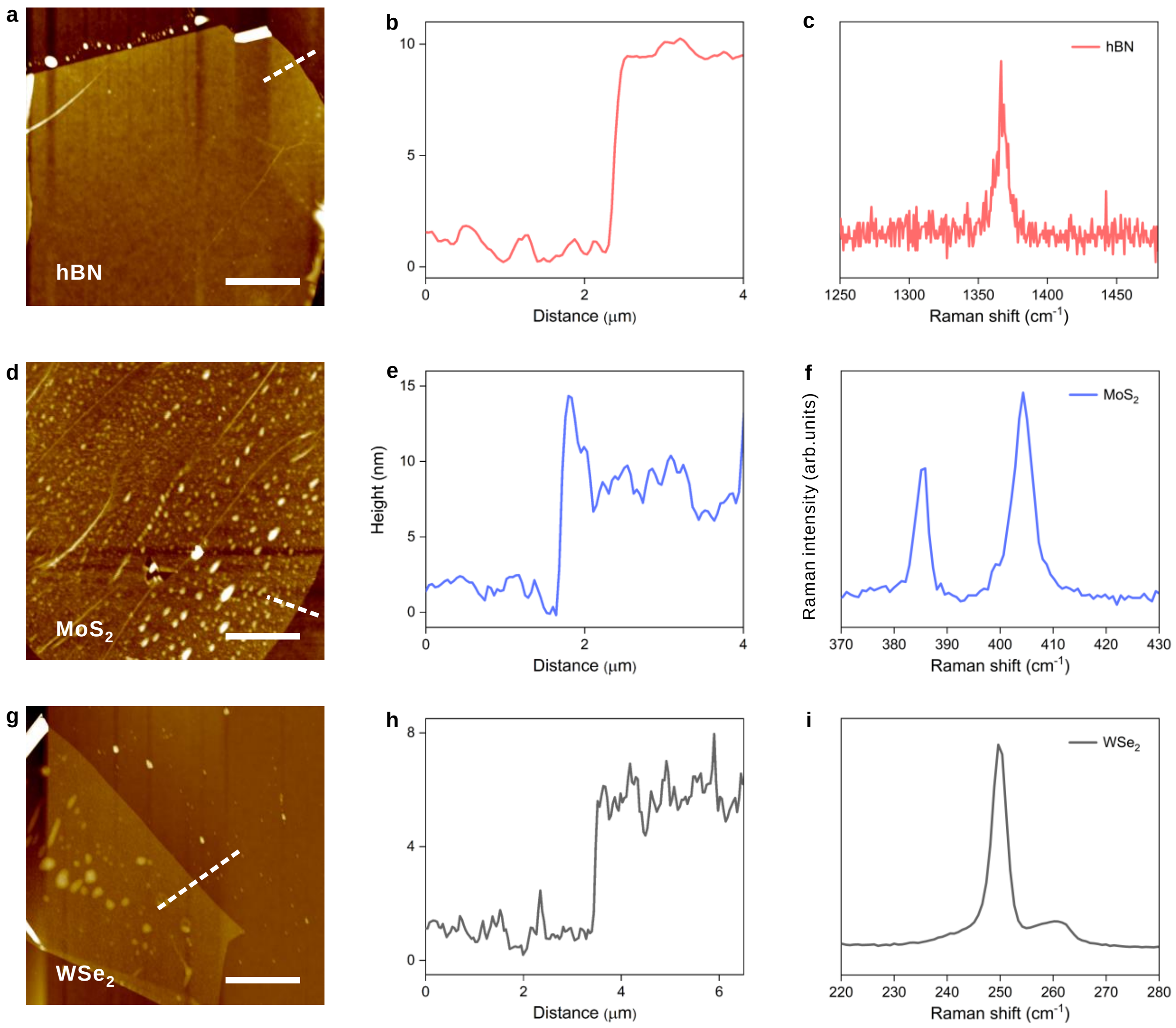


**Supplementary Figure 5: Characterization of $MoS_2$, h-BN and $WSe_2$. a,** hBN thin film characterized by atomic force microscopy (AFM). Scale bar, 5 μm. **b,** The thickness of hBN thin film measured by AFM along the white line marked in (a). **c**, Raman spectrum on the hBN region. **d**, $MoS_2$ thin film characterized by AFM. Scale bar, 5 μm. **e,** The thickness of $MoS_2$ thin film measured by AFM along the white line marked in (d). **f**, Raman spectrum on the $MoS_2$ region. **g**, $WSe_2$ thin film characterized by AFM. Scale bar, 5 μm. **h**, The thickness of $WSe_2$ thin film measured by AFM along the white line marked in (g). **i**, Raman spectrum on the $WSe_2$ region.

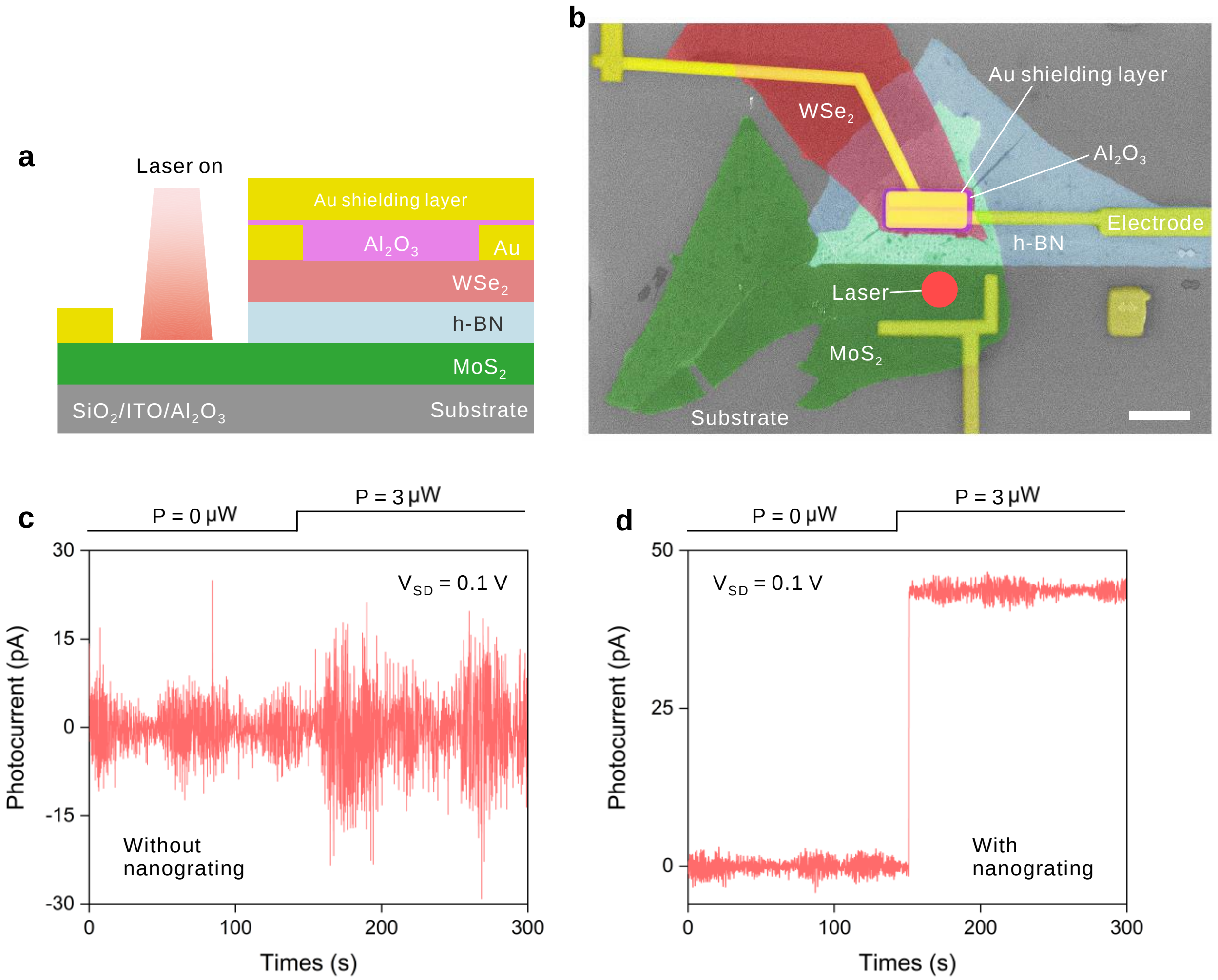


**Supplementary Figure 6: Comparison of photocurrent in 2D PPT with and without Ag nanogratings.** **a**, Schematic of a single 2D PPT without Ag nanogratings. **b**, False-coloured SEM image of a fabricated device without Ag nanograting. The red dot indicates the location of the laser on $MoS_2$. Scale bar, 20 μm. **c**, When the laser is turned on and off, only subtle changes of photocurrent can be detected in the $WSe_2$ channel, indicating that very few electrons in the $MoS_2$ excited by the device without Ag nanograting. **d**, When the laser is turned on and off, a significant change of photocurrent is detected in the $WSe_2$ channel, indicating that a large number of electrons are excited through plasmon decay in devices with Ag nanogratings. The generated electrons are transferred to the right side of the $MoS_2$ floating gate under the photothermoelectric effect and induce a large number of holes in the $WSe_2$ channel.

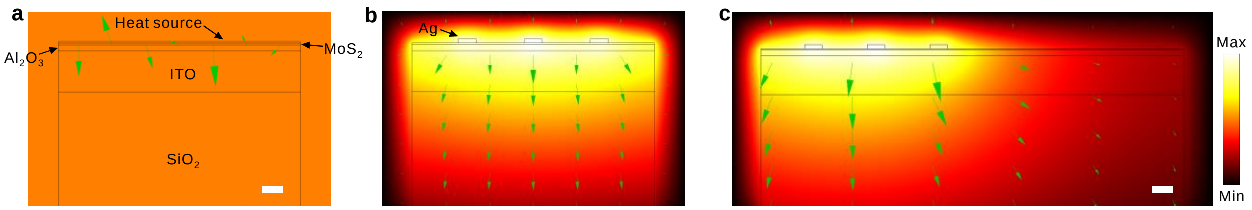


**Supplementary Figure 7: Photothermoelectric effect of 2D PPT with and without Ag nanogratings.** **a-b**, Simulated temperature distribution of the left part of the phototransistor without (a) and with Ag nanograting (b). The colored images indicate the temperature distribution and the green arrows indicate the heat flux. The heat source[2] (heat power density) is written as $Q = \frac{1}{2}\omega \mathrm{Im}(\varepsilon_r)|\mathbf{E}|^2$, where $\omega$ is angular frequency of the light, $\varepsilon_r$ is the relative permittivity of silver, and E is electric field calculated in Fig. 2d. In the absence of the right part of the device, the heat generated by plasmon decay is mainly transmitted downward, resulting in an increase in the temperature of $MoS_2$. Scale bar, 100 nm. **c**, The simulated temperature distribution of the $MoS_2$ floating gate layer of the device extending to the right. The heat generated by the plasmon decay is transmitted to the lower right, but the heat is still mainly concentrated in the Ag nanograting region, resulting in a thermoelectric potential between the left and right sides of the $MoS_2$ floating gate layer. Scale bar, 100 nm.

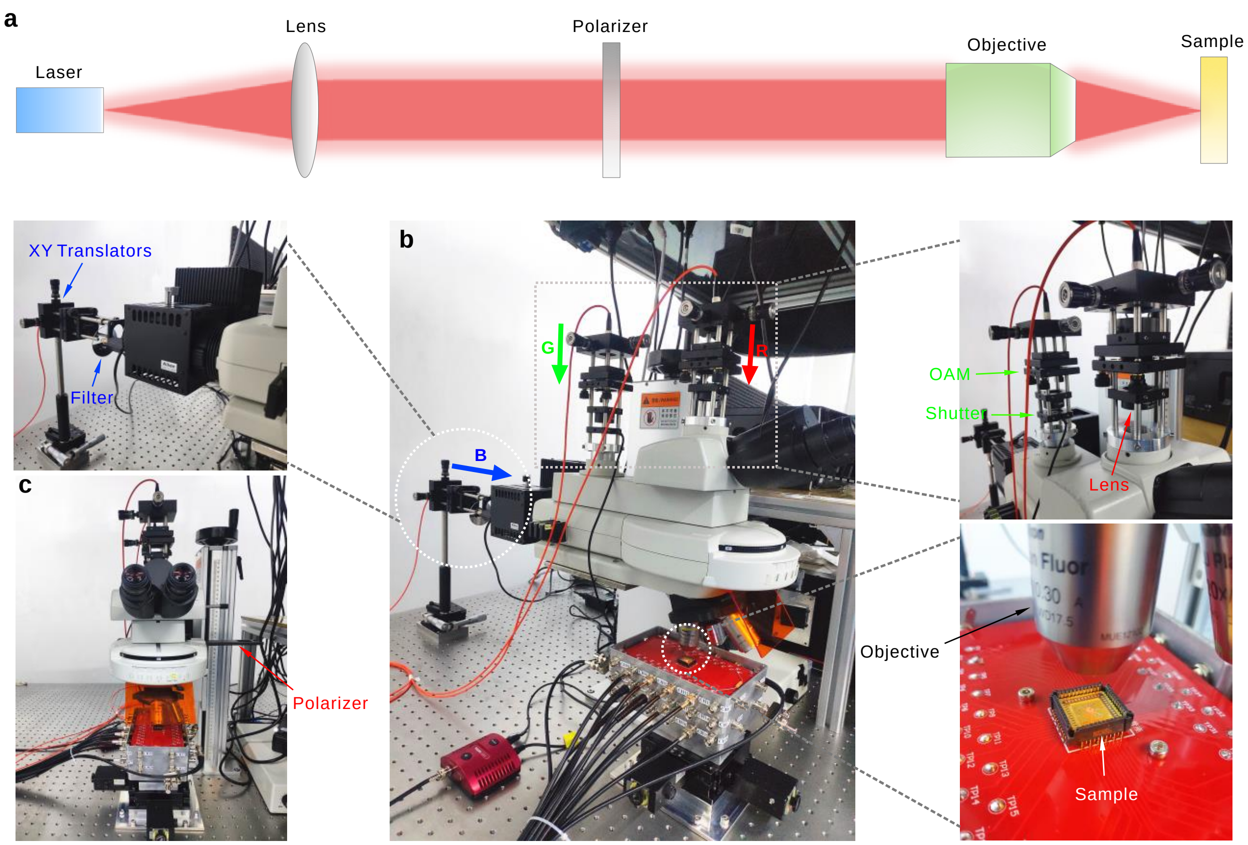


**Supplementary Figure 8: Optical setup.** **a**, Schematic illustration of the optical setup. The polarizer enables that the linearly polarized light (TM polarization) generated by the incident laser is perpendicular to the Ag nanograting. **b**, Photograph of the optical setup. OAM: Optical Adjustable Mount. **c**, Front view photo of the optical setup.

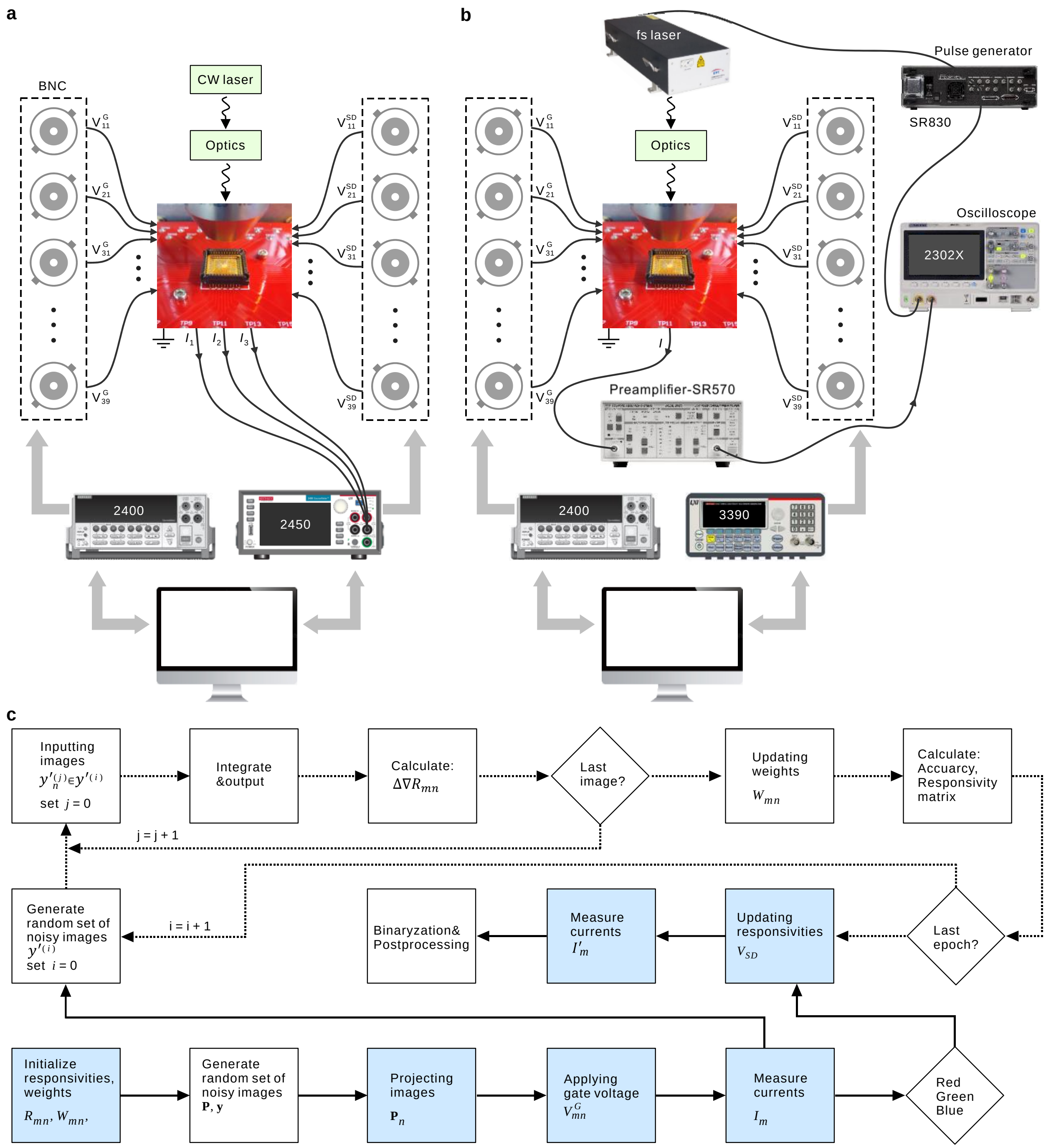


**Supplementary Figure 9: Experimental setup.** **a**, Experimental setup of an AVPRM used as a classifier. CW, continuous wave. Optical setup is shown in Extended Data Fig. 5. **b**, Experimental setup for time-resolved measurements. **c**, Flow chart of AVPRM used for image processing. The blue shaded boxes indicate the ANN PPTA in action. Here, $I'_m = \sum_{n=1}^{N} I'_{mn} = \sum_{n=1}^{N} R'_{mn} P_n$ represents the training current,

which is the output current of the array after being trained in a certain epoch. $R'_{mn}$ is the photoresponsivity after being trained in a certain epoch, and $P_n$ is the incident light power of the $n$th pixel with noise. The output current $I_m$ that makes the activation function $\phi_m(I) = \mathrm{e}^{I_m \xi} / \sum_{k=1}^{M} \mathrm{e}^{I_k \xi}$ of the corresponding output node equal to 1 is defined as the target current. The recognition accuracy is defined as the maximum probability of the output node processed by the activation function after each epoch training, i.e. $\phi(I'_m) \times 100\%$, where $I'_m$ is the training current. The $\Delta = P_n\left(\phi_m(I) - \phi(I'_m)\right)$ here is exactly the difference between the target value and the training value.

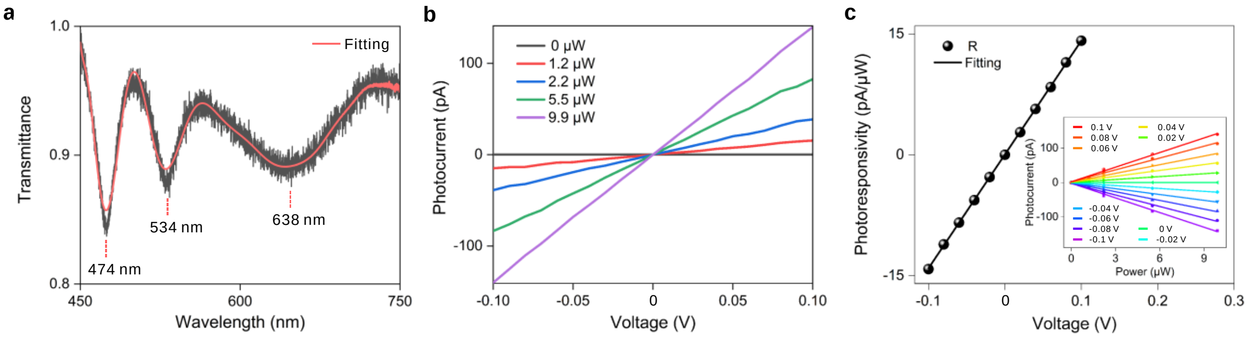


**Supplementary Figure 10: Measurement of optoelectronic characteristics of 2D PPT.** **a**, Experimentally measured normalized transmittance spectra of the WPPs structure on the left side of the device. **b**, $I_{PH}$-$V_{DS}$ curves at different red light powers without any applied gate voltage. **c**, Voltage tunability of the regularized photoresponsivity extracted from (**b**). The inset shows $I_{PH}$ versus P for different $V_{DS}$ values.

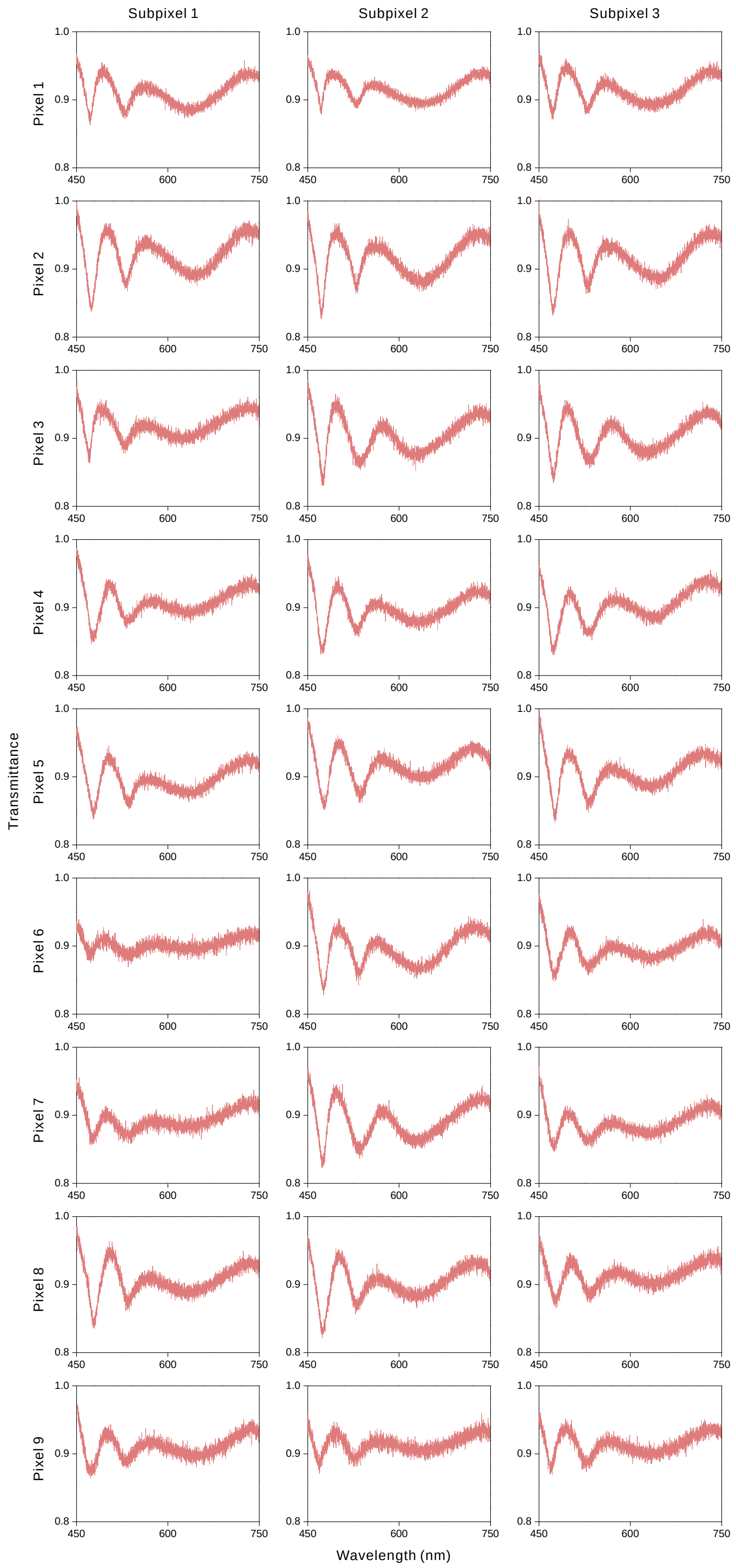


**Supplementary Figure 11: PPTA uniformity.** The measurement of transmission spectra of 27 PPTs indicates that the device has good uniformity.

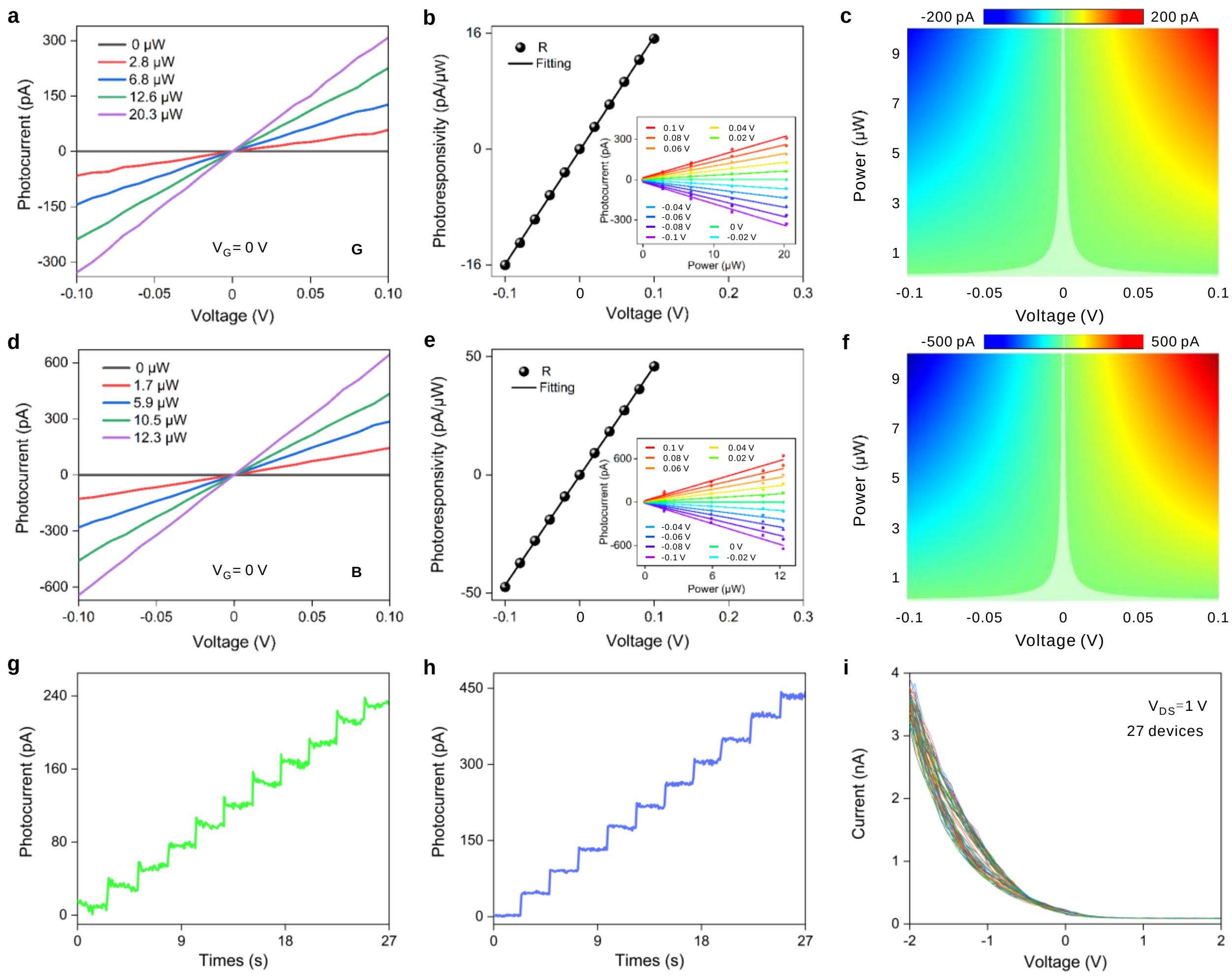


**Supplementary Figure 12: Measurement of optoelectronic characteristics of 2D PPT. a**, $I_{PH}$-$V_{DS}$ curves at different green light powers without any applied gate voltage. **b**, Voltage tunability of the regularized photoresponsivity extracted from (a). The inset shows $I_{PH}$ versus P for different $V_{DS}$ values. **c**, The voltage ($V_{DS}$) tunable photocurrent (green light) corresponding to each gray scale. **d**, $I_{PH}$-$V_{DS}$ curves at different blue light powers without any applied gate voltage. **e**, Voltage tunability of the regularized photoresponsivity extracted from (d). The inset shows $I_{PH}$ versus P for different $V_{DS}$ values. **f**, The voltage ($V_{DS}$) tunable photocurrent (blue light) corresponding to each gray scale. **g-h.** The multi-state photocurrents corresponding to different levels of optical power (gray levels), where the laser wavelengths are 532 nm (g) and 473 nm (h), respectively, and the drain-source voltage is 0.1 V. **i,** The transfer characteristic curves of 27 devices measured under dark conditions at $V_{DS}$ = 1V.

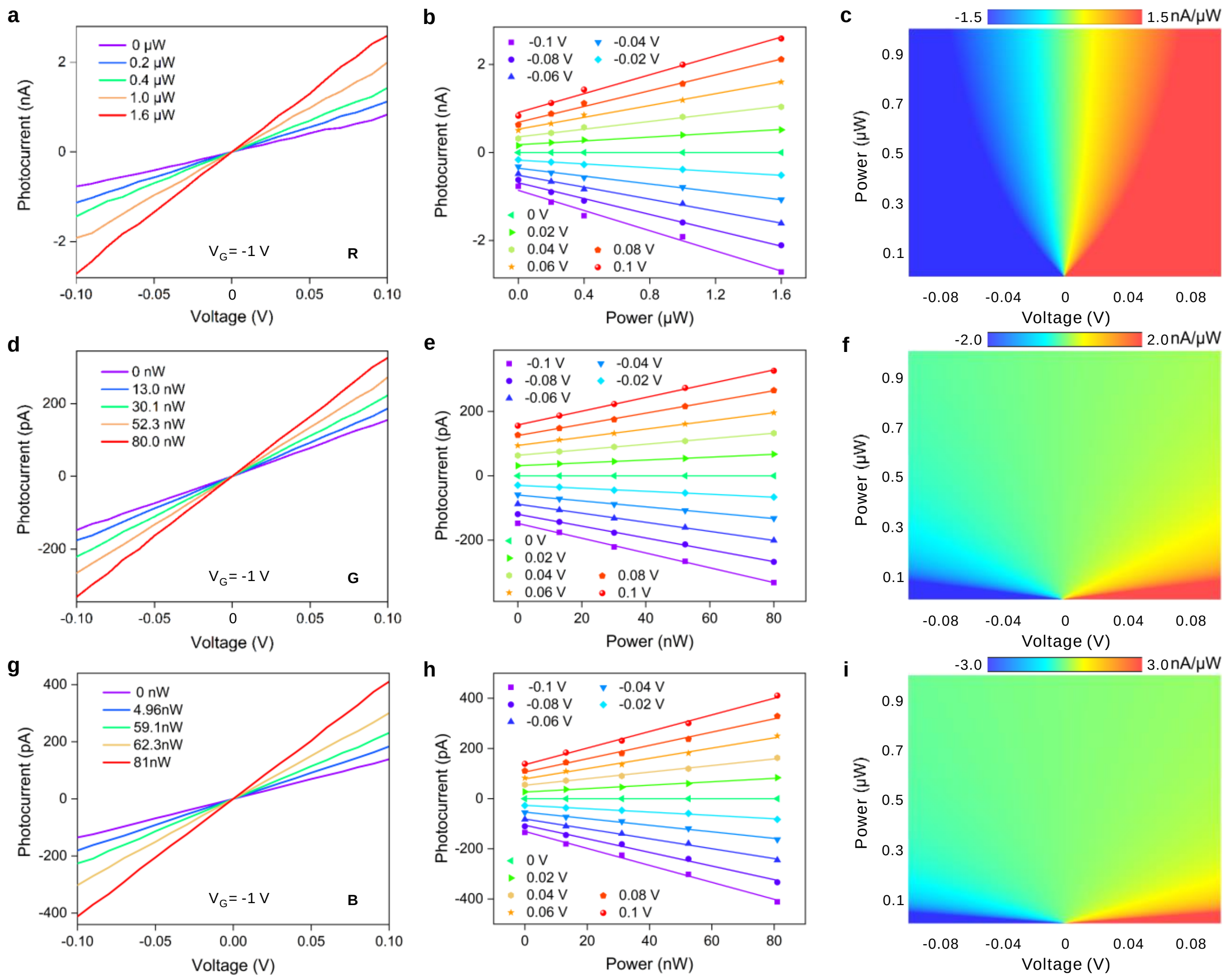


**Supplementary Figure 13: Implementation of PPT photoresponsivity.** **a**, $I_{PH}$-$V_{DS}$ curves at different red light powers with applied gate voltage. **b**, The relationship between $I_{PH}$ and P under different $V_{DS}$ values extracted from (**a**). **c**, The voltage ($V_{DS}$) tunable red light regularized photoresponsivity corresponding to each gray scale. **d**, $I_{PH}$-$V_{DS}$ curves at different green light powers with applied gate voltage. **e**, The relationship between $I_{PH}$ and P under different $V_{DS}$ values extracted from (**d**). **f**, The voltage ($V_{DS}$) tunable green light regularized photoresponsivity corresponding to each gray scale. **g**, $I_{PH}$-$V_{DS}$ curves at different blue light powers with applied gate voltage. **h**, The relationship between $I_{PH}$ and P under different $V_{DS}$ values extracted from (**g**). **i**, The voltage ($V_{DS}$) tunable blue light regularized photoresponsivity corresponding to each gray scale.

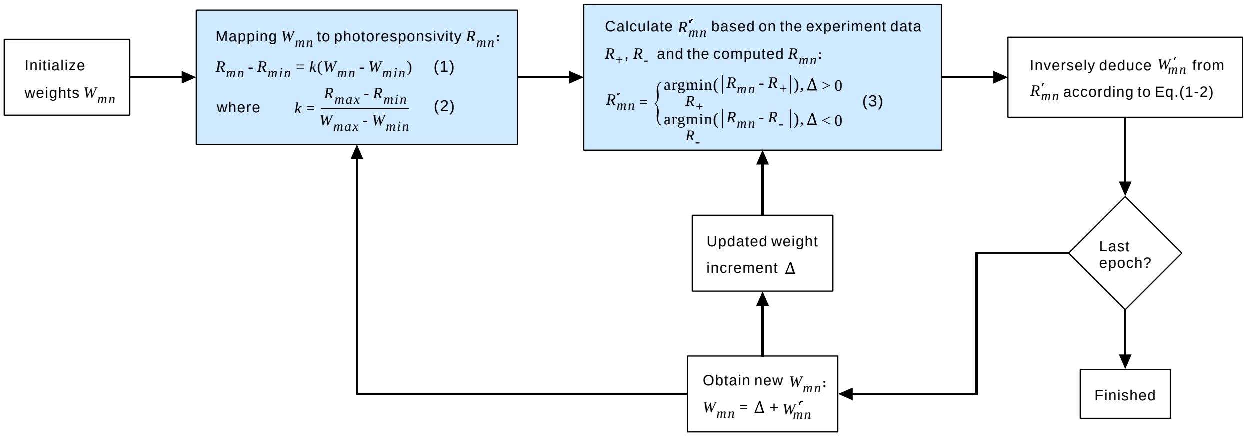


**Supplementary Figure 14: The training processes of the ANN with experimental photoresponsivity.** The ANN is trained off-line according to the experimental photoresponsivity curve in Supplementary Fig. 10c. $R_+$ represents the positive potentiation part of the photoresponsivity curve, while $R_-$ represents the negative depression part of the photoresponsivity curve. The responsivities were updated by backpropagation[3].

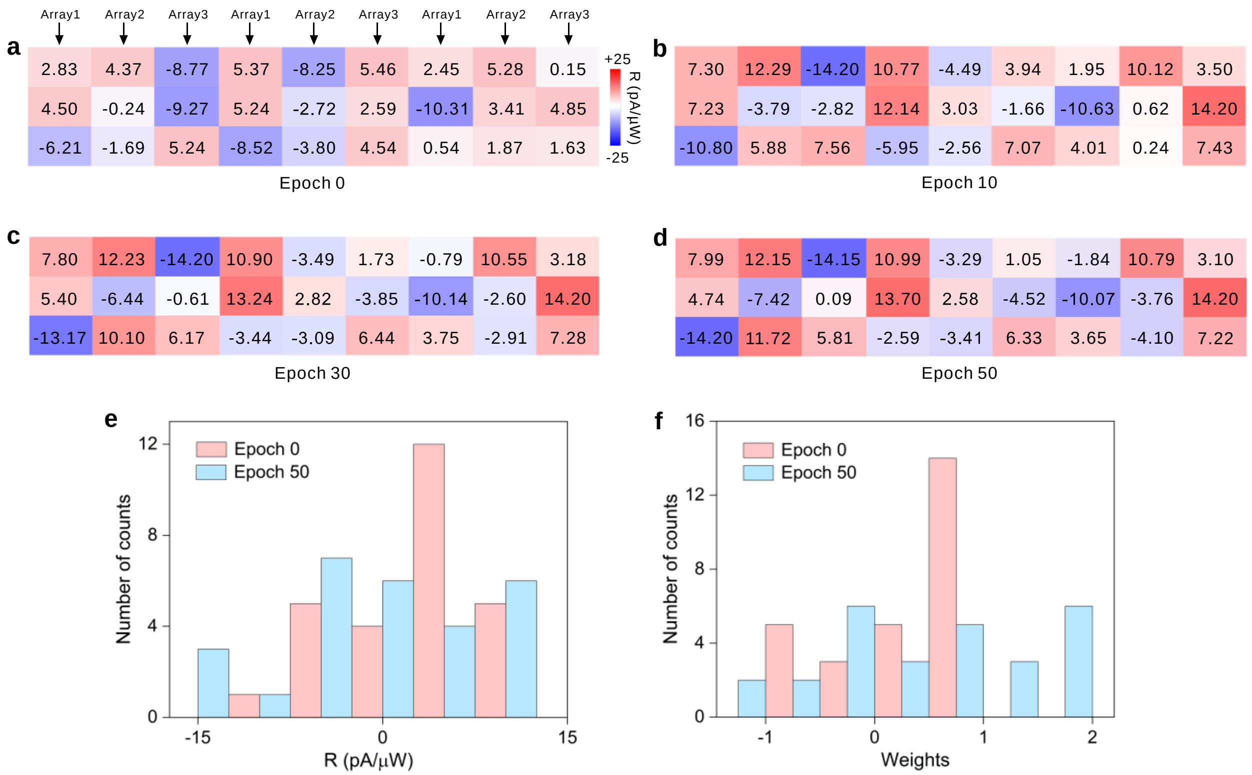


**Supplementary Figure 15: Photoresponsivity and weight distributions of the array. a-d**, The corresponding photoresponsivity of the array after 0 (a), 10(b), 30(c), and 50(d) training epochs, respectively. **e-f**, Photoresponsivity (e) and weight (f) distributions before (initial) and after (final) training.

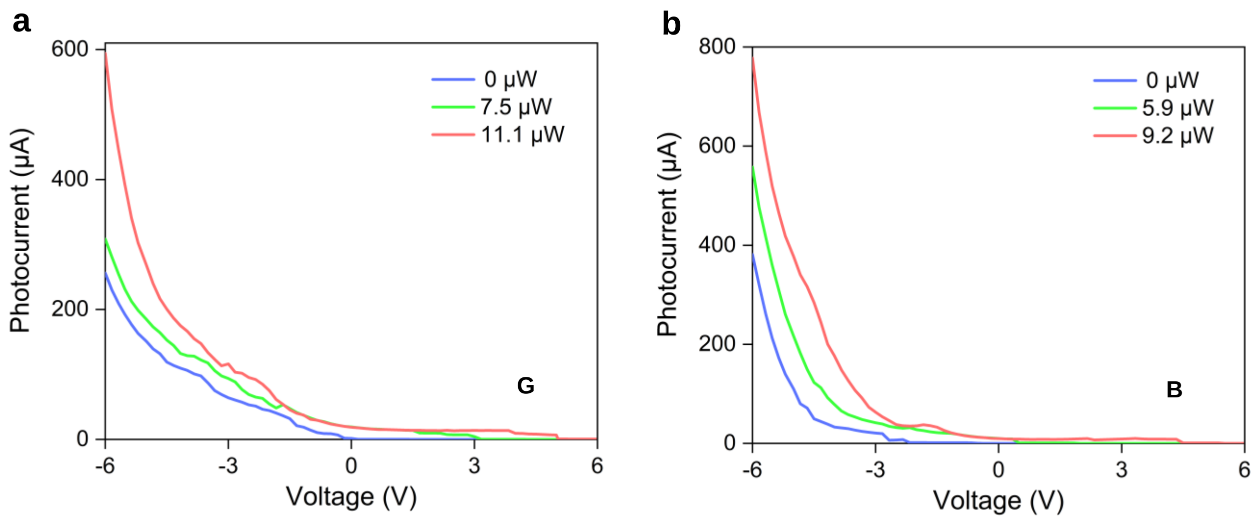


**Supplementary Figure 16: Transfer characteristic curves. a-b,** The transfer characteristic curves of the devices with green (a) and blue (b) light measured under different P values at $V_{DS}$ = 1 V, respectively.

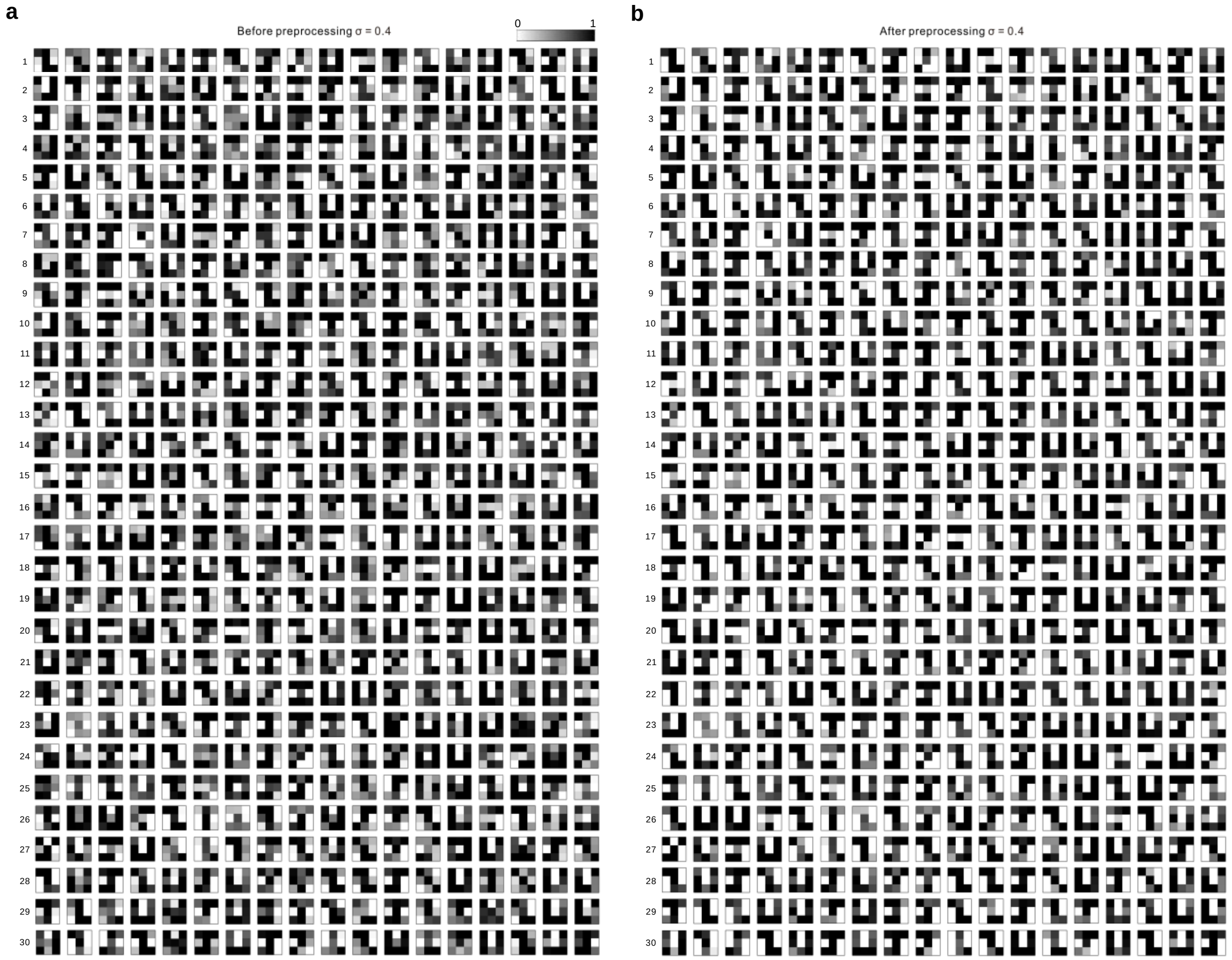


**Supplementary Figure 17: Training datasets. a**, **b**, Dataset with noise level σ = 0.4 for classifier training with (**a**) and without (**b**) pre-processing.

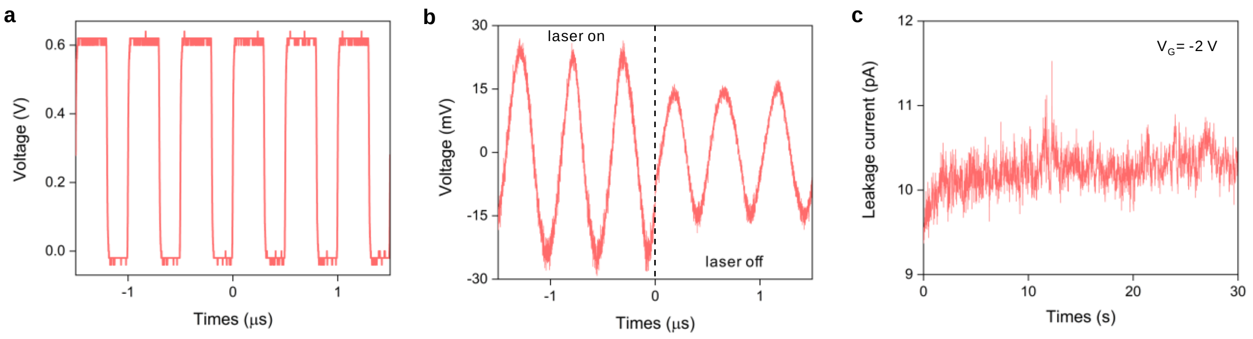


**Supplementary Figure 18: Time-resolved measurements.** **a**, Electrical pulse for synchronously triggering laser and drain-source current measurement. **b**, The drain-source voltage signal when the laser is turned on or off after preamplifier conversion. **c**, Leakage current when side gate voltage is applied to assist measurement.

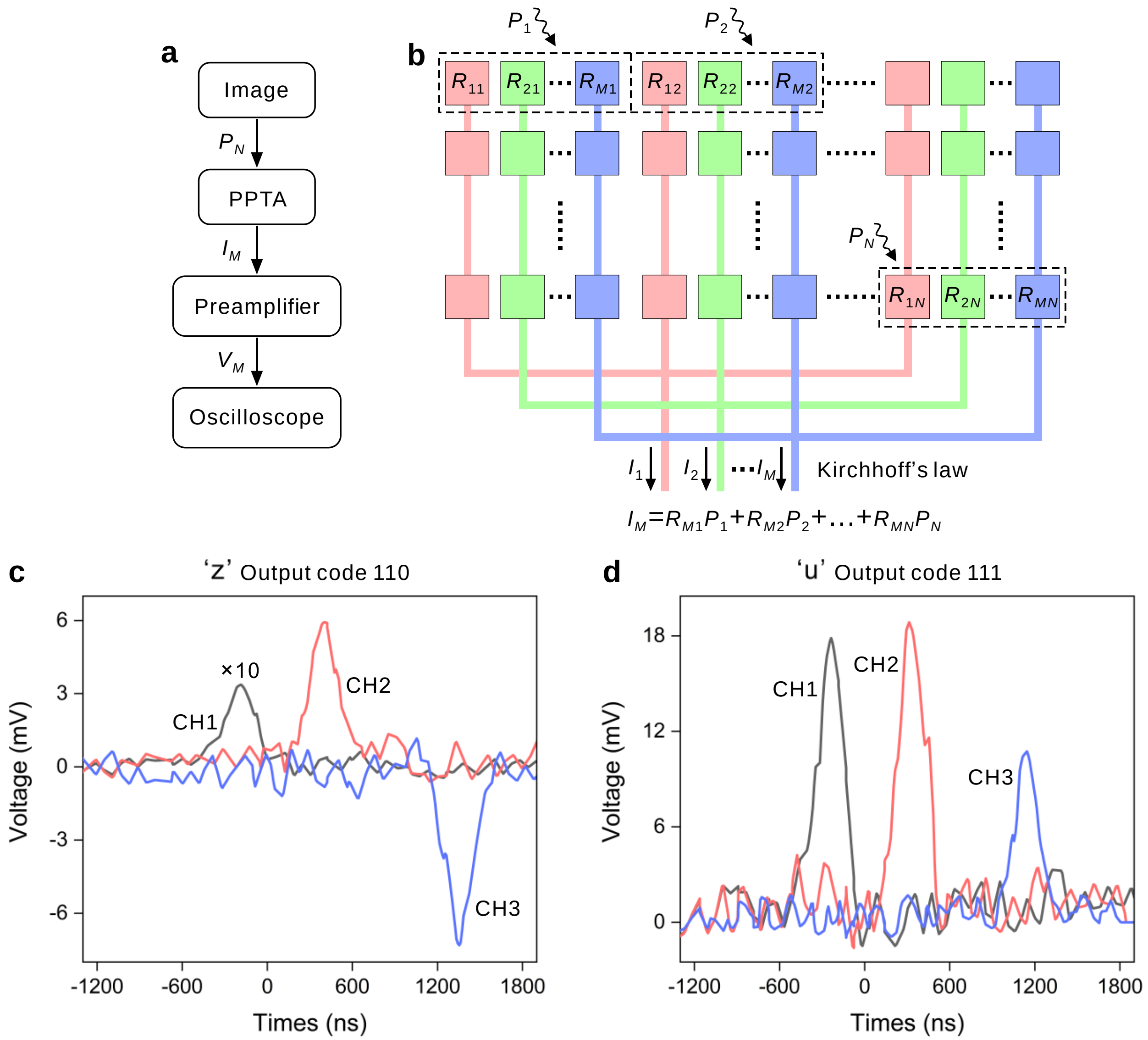


**Supplementary Figure 19: Ultrafast image recognition. a**, Flowchart for ultrafast image recognition. $P_N$ represents the ultrafast signal incident on each pixel, $I_M$ is the total output current of all $M$-th subpixels, and $V_M$ is the voltage obtained by converting and amplifying $I_M$ through a preamplifier. **b**, Schematic diagram of a PPTA used to perform multiply-and-accumulation (MAC) operations in a neural network. $R_{MN}$ is the regularized photoresponsivity of the subpixel. **c, d,** Projection of two different letters, 'z' and 'u', with a duration of 500 ns, leads to distinct output voltage codes 110 (c) and 111 (d) for the three channels. CH represents the output channel. The encoding rule for output voltage are the same as in the maintext, with positive signals marked as 1 and negative signals marked as 0.

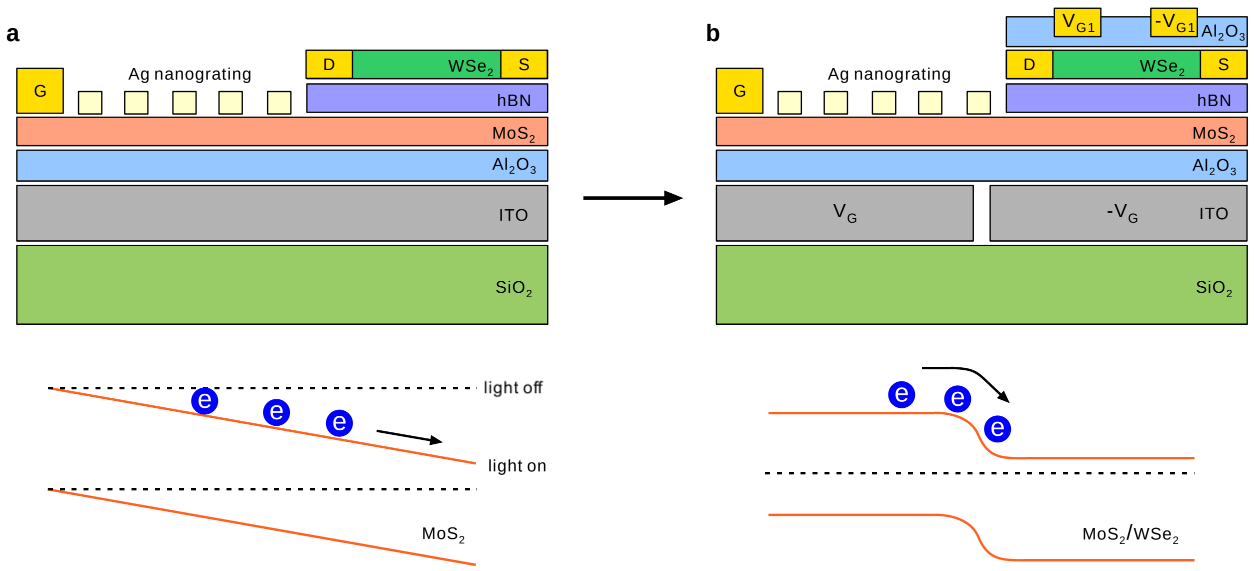


**Supplementary Figure 20: Discussion on the limited speed of device operation. a,** Schematic of the PPT. The bottom of the figure shows the energy band diagram of $MoS_2$ under light on and light off conditions, respectively. Under the condition of light on, the thermoelectric potential generated by the the plasmon effect tilts the energy band, leading to the transport of hot electrons from the hot end to the cold end. The establishment of the thermoelectric potential and the transport of hot electrons prolongs the whole process. **b,** Schematic of the improved PPT. In this device, the bottom ITO and the top shielding layer are made into splitting electrodes respectively. As shown in the energy band diagram at the bottom, $MoS_2/WSe_2$ energy band can be modulated into PN junction energy band after applying positive/negative gate voltage to the splitting electrode. The hot electrons generated by plasmon decay and the induced carriers in the channel will migrate rapidly under the effect of the energy potential, thus accelerating the whole process.

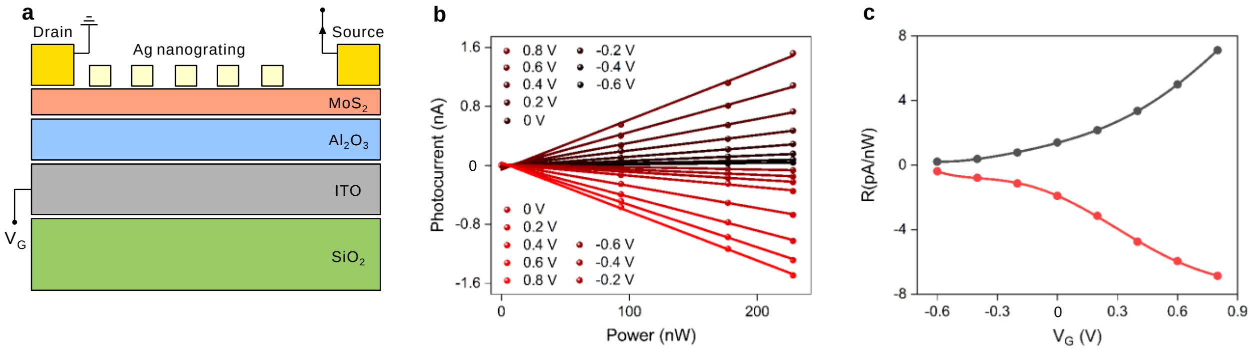


**Supplementary Figure 21: Plasmon-enhanced photodetector with adjustable photoresponsivity. a,** Schematic of the plasmon-enhanced photodetector. The device operates under short-circuit conditions and sets the photoresponsiveness by supplying a voltage to the ITO bottom gate electrode against $V_G$. The device is operated under short-circuit conditions and the photoresponsivity is set by supplying a voltage $V_G$ to the ITO bottom gate electrode. The thickness of each material in the device is consistent with the maintext. **b,** The short-circuit photocurrent measured by modulating the bottom ITO gate voltage under different optical power, where the positive/negative photocurrents were obtained by switching the polarity of the source/drain controlled by an external circuit. **c,** Voltage tunability of the photoresponsivity extracted from (b). The weight (photoresponsivity) of the ANN training process can be obtained by adjusting the gate voltage.

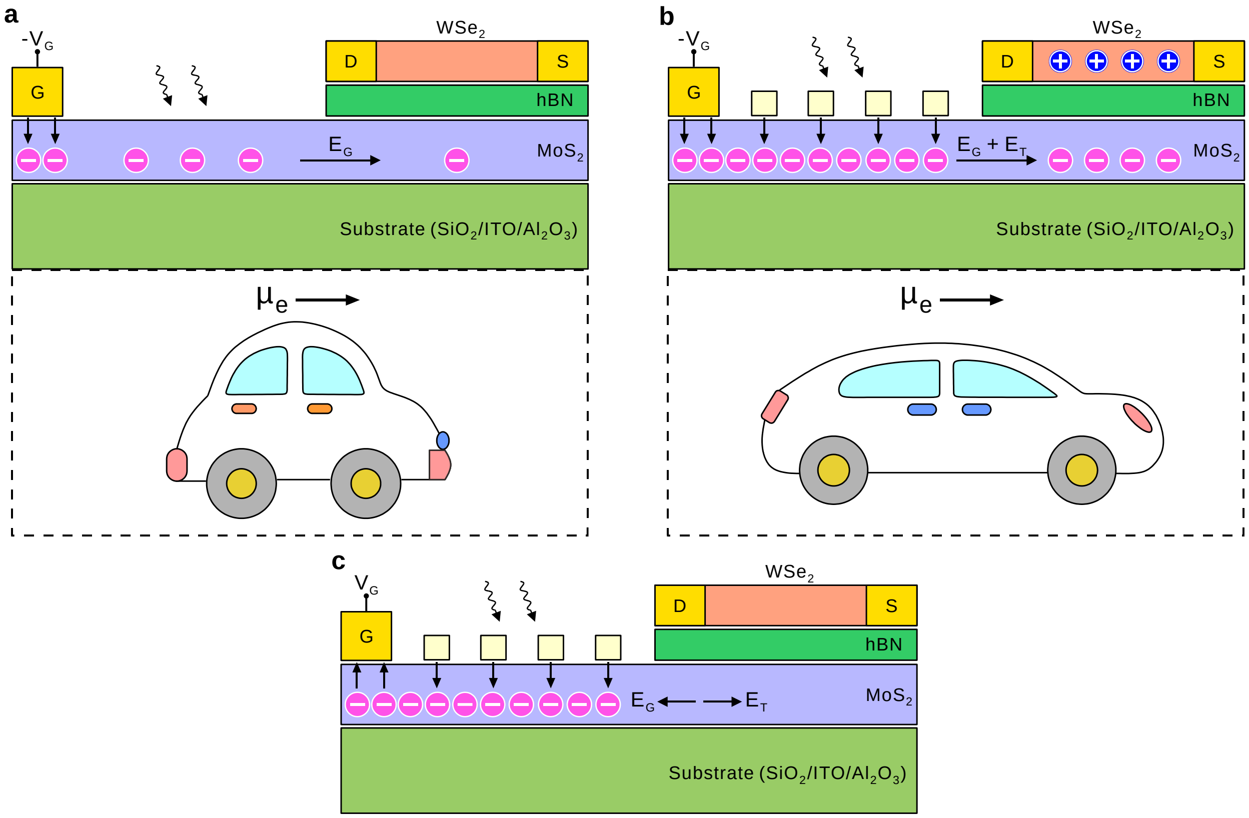


**Supplementary Figure 22: Schematic of the mechanism of the 2D PPT device with ultra-high DR. a,** Schematic analysis of electron concentration ($n$), mobility ($\mu_e$), and drag potential ($E_G$) in device without nanograting. In this case, only a small number of electrons are dragged to the right side of the $MoS_2$ floating gate, hardly inducing a charge in $WSe_2$ channel, which makes the current difficult to be measured. The lower part of the graph visualizes the mobility ($\mu_e$) as the acceleration ability of an ordinary car. **b,** Schematic analysis of electron concentration ($n$), mobility ($\mu_e$), and drag potential ($E_G + E_T$) in the PPT device with nanograting. In this case, a sufficient amount of electrons are dragged to the right side of the $MoS_2$ floating gate, which induces enough charge in $WSe_2$ channel, leading to a maximum current ($I_{max}$) that can be measured. The lower part of the graph visualizes the mobility ($\mu_e$) as the acceleration ability of a supercar. **c,** Schematic of the PPT device in which electrons cannot be dragged to the right side of $MoS_2$ floating gate under positive gate potential modulation. In this case, almost no electrons can be dragged to the right side of the $MoS_2$ floating gate, which also cannot induce charges in the $WSe_2$ channel, resulting in a measurable minimum current ($I_{min}$).

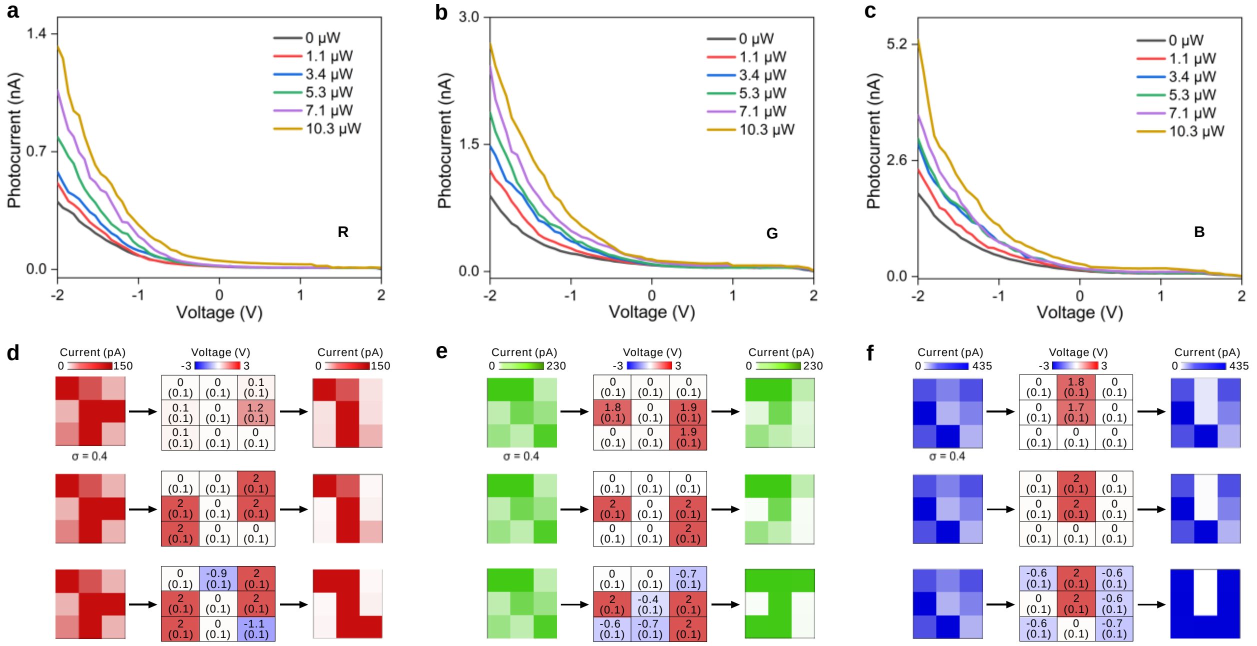


**Supplementary Figure 23: Transfer characteristic curve and its application in image pre-processing process. a-c,** The transfer characteristic curves of the devices with red (a) green (b) and blue (c) light measured under different P values at $V_{DS}$ = 0.1 V, respectively. **d-f,** The pre-processing process corresponds to the red letter ‘z’ (d), the green letter ‘j’ (e) and the blue letter ‘u’ (f), respectively. The left column represents the image with Gaussian noise (σ = 0.4) added before pre-processing, the middle column represents the modulation voltage required for pre-processing, and the right column represents the image after pre-processing. Here, the modulation voltage in parentheses is the drain-source voltage $V_{DS}$.

**Supplementary Table 1 Summary of the performance of individual plasmonic phototransistor.**

| Type | Mobility: light on/off ($cm^2 V^{-1} s^{-1}$) | On/off ratio | t (ns) | R (pA/μW) | Leakage current (pA) | Dark current (pA) |
|---|---|---|---|---|---|---|
| Red | 154 / 23.3 | ~ $1 \times 10^8$ | 500 | -14/+14 | ~ 10.2 | ~ 1.5 |
| Green | 233 / 23.4 | ~ $1 \times 10^8$ | 500 | -16/+16 | ~ 10.2 | ~ 3.1 |
| Blue | 273 / 23.6 | ~ $1 \times 10^9$ | 500 | -47/+47 | ~ 10.2 | ~ 2.3 |

**t**: response time. **R**: Photoresponsivity.

**Supplementary Table 2 Comparison of the proposed neuromorphic device with previous report.**

| Pixel structure | Integrated function | OSR | E | t | DR | CC |
|---|---|---|---|---|---|---|
| [6]A hBN/$WSe_2$ synaptic device + hBN/$WSe_2$ photodetector | Sensing + postprocessing (pattern recognition) | N/A | 66-532 fJ | 10 ms | N/A | Yes |
| [7]A $WSe_2$ photodiode | Sensing + postprocessing (classify/encode images) | Yes | N/A | 40 ns | N/A | Yes |
| [8]A resistive pressure sensor + perovskite-based photodetector + hydrogel-based ionic cable and a synaptic transistor | Sensing (visual-haptic fusion) + postprocessing (pattern recognition) | N/A | N/A | 1 s | N/A | N/A |
| [9]A $MoS_2$-pV3D3 phototransistor | Sensing + preprocessing | N/A | N/A | 0.5 s | N/A | Yes |
| [10]A synaptic device with a structure of Pd/$MoO_x$/ITO | Sensing + preprocessing (background noise reduction) | N/A | N/A | 200 ms | N/A | Yes |
| [11]A $MoS_2$ phototransistor + UVO treatment | Sensing + preprocessing (Scotopic/Photopic adaptaton) | N/A | N/A | N/A | 199 dB | Yes |
| A $MoS_2$/hBN/$WSe_2$ plasmonic phototransistor | Sensing + preprocessing (image contrast enhancement) | Yes | $2.4 \times 10^{-17}$ J | 500 ns | 180 dB | Yes |

| | | | | | | |
|---|---|---|---|---|---|---|
| | + postprocessing (image recognition) | | | | | |

**DR** stands for dynamic range and can be used to enhance image contrast (preprocessing) by adjusting DR. **E**: energy consumption. **t**: response time. **OSR**: On-site recognition. **CC**: CMOS compatibility.

**Supplementary Note 1. The reason and mechanism of the device with ultra-high DR**

For the device without nanograting, as shown in Supplementary Fig. 22a, carriers are generated under light due to the photoresponsive properties of the 2D $MoS_2$ itself. After applying the gate voltage -$V_G$, a few carriers are dragged to the right end after undergoing a series of processes (e.g., electron-electron scattering, electron-phonon coupling), making it almost difficult for such a small number of carriers to induce charges in the $WSe_2$ channel. According to $\mu_e = e\tau/m^*$, where $e$ is the elementary charge, $\tau$ is the relaxation time, and $m^*$ is the effective mass, it is clear that nothing in this process can cause the carrier mobility $\mu_e$ to be increased. For the PPT device with nanograting, as shown in Supplementary Fig. 22b, in addition to the carriers generated under illumination due to the photoresponsive properties of the $MoS_2$ itself, there is also a portion of hot electrons generated by plasmon decay. In summary, the carrier concentration in the $MoS_2$ floating gate increases compared to the case in Supplementary Fig. 22a.

On the other hand, the dephasing of plasmon can also cause an increase in electron temperature and lattice temperature. The relaxation time of hot electrons is proportional to $\Delta T$, $\Delta T$ is the temperature increase caused by the pump laser[4]. Therefore, the mobility of electrons is improved due to the increase in temperature. If the electron mobility can be compared to the acceleration ability of a car, as shown in the lower part of Supplementary Fig. 22, then the difference in electron mobility between the two structures is similar to the difference in acceleration ability between an ordinary car and a supercar. In addition, the increase in lattice temperature also causes a temperature difference between the two ends of $MoS_2$, which

leads to the formation of thermoelectric potential[5]. After applying the gate voltage -$V_G$, the carriers would be transported toward the right end of $MoS_2$ under the joint drag of the gate potential $E_G$ and the thermoelectric potential $E_T$. As a result, the electron concentration ($n$), mobility ($\mu_e$), and drag electric field ($E_G + E_T$) are greatly enhanced in devices with nanograting, which leads to a significant increase in electron transport to the right end of $MoS_2$. The accumulation of electrons on the right side of $MoS_2$ induces a sufficient charge in the $WSe_2$ channel, and the maximum current ($I_{max}$) can be measured by modulating the gate voltage and laser power. On the contrary, as shown in Supplementary Fig. 22c, when a positive gate voltage $V_G$ is applied to generate a potential $E_G$ much greater than the thermoelectric potential $E_T$, almost no electrons can be transported to the right side of $MoS_2$ floating gate, and naturally almost no charge can be induced in the $WSe_2$ channel. In this case, the minimum current ($I_{min}$) can be measured. Here, the introduction of nanogratings into the PPT device increases the difference between $I_{max}$ and $I_{min}$, and according to DR = 20 × log[$I_{max}$ / $I_{min}$] (dB), ultra-high DR can be obtained.

**Supplementary Note 2. An analysis on the performance of the individual plasmonic phototransistor.** According to the data presented in Fig. 3h, the mobility of the device at maximum optical power incidence is 154 $cm^2\ V^{-1}\ s^{-1}$, which can be extracted using the expression $\mu = [\mathrm{d}I_D/\mathrm{d}V_G]\times[L/(WCV_{DS})]$, where $L$ = 1.9 μm is the channel length, $W$ = 4.2 μm is the channel width, and $C$ is the capacitance between the channel and the gate per unit area ($C = \varepsilon_0\varepsilon_r/d_{\mathrm{hBN}}$; $\varepsilon_r = 3.5$; $d_{\mathrm{hBN}} = 10$ nm). Similarly, the mobility of the device under green and blue light incidence with maximum optical power is 233 $cm^2\ V^{-1}\ s^{-1}$and 273 $cm^2\ V^{-1}\ s^{-1}$, respectively, which can also be derived from the data presented in Supplementary Fig. 16. It is clear that the mobility of 2D materials has been greatly improved. On the one hand, the increase in mobility could be due to the suppression of Coulomb scattering by the deposited dielectric $Al_2O_3$. On the other hand, the dephasing of plasmon generated by the Ag nanograting via photoexcitation leads to an increase in electron temperature and lattice temperature. Since the relaxation time of hot electrons is proportional to the

temperature increment $\Delta T$, the relaxation time of hot electrons increases. According to $\mu_e = e\tau/m^*$, where $e$ is the elementary charge, $\tau$ is the relaxation time, and $m^*$ is the effective mass, the carrier mobility is greatly improved. As shown in Supplementary Fig. 10a, the normalized transmittance spectrum of the WPPs structure in the device indicates that the absorption rate of the device increases as the wavelength decreases. That is, at the same optical power, the nanostructure in the device absorb blue light the most, followed by green light and finally red light. Therefore, the mobility of the device under green light incidence is maximum. Conversely, in the absence of light, the temperature of the nanostructure in the device remains unchanged, resulting in a significant decrease in mobility.

For the device with nanograting, under illumination, the dephasing of plasmons not only increases the device temperature, but also leads to a significant increase in electron concentration $n$ in the device. According to $\sigma = ne\mu$, it is evident that the conductivity $\sigma$ of the device is enhanced, and the on-state current is subsequently increased, ultimately resulting in a current on/off ratio exceeding $1 \times 10^9$. It is also due to the introduction of nanogratings that the photoresponsivity of the device has been greatly improved. Although the entire process from the generation of hot electrons by plasmon decay to the injection into $MoS_2$ is accomplished on a sub-nanosecond level, the transfer of the hot electrons and the establishment of the thermoelectric potential prolong the entire process. In order to illustrate the high-speed capabilities of PPTA, we carried out measurements by employing a 500 ns pulsed laser source and an electric pulse source with synchronous triggering. As shown in Supplementary Fig. 19, we plot the electric output pulses, with different output codes representing different image types, which demonstrate the correct pattern classification within ~500 ns. The dielectric h-BN between the channel $WSe_2$ and the gate $MoS_2$ derived from bulk source materials by a mechanical peel-transfer method. The weak leakage current indicates that BN has good uniformity, insulation, and clean interface. Meanwhile, the negligible dark current indicates the absence of impurities and interface charges at the heterostructure interface, which is also verified in the scanning transmission electron microscope image shown in Fig. 1g.

## Supplementary References


1. Wang, C.-Y. et al. Gate-tunable van der Waals heterostructure for reconfigurable neural network vision sensor. *Sci. Adv.* **6**, eaba6173 (2020).
2. Chalabi, H., Schoen, D. & Brongersma, M. L. Hot-Electron Photodetection with a Plasmonic Nanostripe Antenna. *Nano Lett.* **14**, 1374-1380 (2014).
3. Rumelhart, D.E., Hinton, G.E. & Williams, R.J. Learning representations by back-propagating errors. Nature 323, 533-536 (1986).
4. Hartland, G. V. Optical studies of dynamics in noble metal nanostructures. *Chem. Rev*. **111**, 3858-3887 (2011).
5. Buscema, M. et al. Large and tunable photothermoelectric efect in single-layer $MoS_2$. *Nano Lett.* **13**, 358-363 (2013).
6. Seo, S. et al. Artificial optic-neural synapse for colored and color-mixed pattern recognition. *Nat. Commun*. **9**, 5106 (2018).
7. Mennel, L. et al. Ultrafast machine vision with 2D material neural network image sensors. *Nature* **579**, 62-66 (2020).
8. Wan, C. et al. An artificial sensory neuron with visual-haptic fusion. *Nat. Commun*. **11**, 4602 (2021).
9. Choi, C. et al. Curved neuromorphic image sensor array using a $MoS_2$-organic heterostructure inspired by the human visual recognition system. *Nat. Commun.* **11**, 5934 (2020).
10. Zhou, F. et al. Optoelectronic resistive random access memory for neuromorphic vision sensors. *Nat. Nanotechnol*. **14**, 776-782 (2019).
11. Liao, F. et al. Bioinspired in-sensor visual adaptation for accurate perception. *Nat. Electron.* **5**, 84-91 (2022).
